# A new model for general practice-led, regional, community-based, memory clinics

**DOI:** 10.1186/s12875-022-01829-1

**Published:** 2022-09-20

**Authors:** Rebecca Disler, Amy Pascoe, Holly Anderson, Ewa Piejko, Adel Asaid, Peter Disler

**Affiliations:** 1 Respiratory Research@Alfred, Department of Immunology and Pathology, Central Clinical School, Monash University, Level 4 The Alfred Centre, 99 Commercial Road, Melbourne , Vic 3004 Australia; 2 Department of Rural Health, Faculty of Medicine, Dentistry and Health Sciences, University of Melbourne, Parkville, VIC 3010 Australia; 3 St Anthony Memory Service, Strathfieldsaye , VIC Australia; 4 School of Rural Health, Monash University, Building 20/26 Mercy St, Bendigo, VIC Australia

**Keywords:** Dementia, Dementia screening, Primary care, General practice, Models of care

## Abstract

**Background:**

Dementia is a major international health issue with high impact on the patient, relatives, and broader society. Routine screening for dementia is limited, despite known benefit of early detection and intervention on quality of care and patient outcomes. Screening is particularly limited in rural and regional areas, despite high burden and projected growth of dementia in these populations. The current study aimed to implement a new general practitioner (GP) led, multidisciplinary, model of care providing dementia detection and referral pathway to a community-based specialist clinic across six regional general practices.

**Methods:**

Cross-sectional analysis of dementia screening and referral characteristics in the St Anthony Family Medical Practices group based in the regional area of Loddon-Mallee, Victoria. Data were collected on demographics and relevant medical history. Cognitive state was assessed using the Mini-Mental State Examination (MMSE), GP Assessment of Cognition (GPCog), and Geriatric Depression Scale (GDS). Referrals and referral outcomes were recorded for geriatrician, psycho-geriatrician, or both.

**Results:**

Eight hundred and eighteenth patients over 65 years were screened, accounting for approximately 24.2% of 65 and over presentations for the practice network. Of those screened, 68.9% were indicated for referral and 30.3% of these were successfully referred. Of the indicated patients who received referrals, 34.2% declined. Many who declined referral had intermediate scores on the cognitive assessments utilized.

**Conclusion:**

Standardised models of care, integrated within community services, are necessary to improve access to early detection, referral and quality management of dementia. The St Anthony Memory Service model will be invaluable in informing future service development, and in particular the development of services for people living with dementia in rural and regional communities.

**Table:** 

The Known • Dementia is a major international health issue, which is estimated to affect one in ten Australians over 65, and the prevalence expected to double by 2050 • Despite the positive impact of early detection and intervention on quality of care and patient outcomes, routine screening is limited, particularly in regional and rural communities
The New • A new model of care, that incorporates training and early dementia detection screening and referral pathway to a community-based specialist clinic, has been implemented and assessed • Of 818 patients screened, 68.9% were indicated for referral and 30.3% of these were successfully referred. Many who declined referral had intermediate scores on the cognitive assessments utilized
The Implications • Primary care-based routine screening may help identify early signs of cognitive decline and generate referrals for specialist management • Further work is needed to target hesitancy in patients with intermediate symptoms

## Background

Dementia is a major international health issue with high impact on the patient, relatives, and broader society. In Australia, dementia is a National Health Priority Area with an estimated 386,200 Australians currently living with dementia and with projections of over 800,000 affected by 2058 [1]. Prevalence is greatest in rural and regional areas (1.2% vs 1.1%), with this divide projected to broaden over the coming decades [2]. Increased prevalence in rural and regional areas is thought to relate to the higher prevalence of risk factors such as hypertension, smoking, obesity, type II diabetes, and the higher proportion of people aged over 75 years [3].

Routine screening for dementia is limited, despite known benefit of early detection and intervention on quality of care and patient outcomes, this again particularly evident in regional and rural communities [4]. Dementia healthcare services have become increasingly managed by specialist services, with the *World Alzheimer Report 2016* calling for primary and community care services to play a more prominent role in diagnosis and management [5]. The need for timely diagnosis, better diagnostic accuracy, and post-diagnosis management and support is essential for patients and their families [6, 7], and has been reinforced by the recent Australian dementia practice guidelines [8].

General Practitioners (GPs) and Practice Nurses (PNs) are well placed to participate in early, timely dementia diagnosis, and for many patients GPs are the first point of contact when symptoms present [9–11]. Despite this, up to 65% of people who were found to have cognitive deficits on standardized testing in American primary care had not been previously considered to have dementia by their treating medical practitioners [12].

Memory clinics are specialised, collaborative services with streamlined diagnostic and post-diagnostic support pathways [13]. The majority of memory clinics are based in tertiary-hospitals and metropolitan areas. Memory clinic capacity is also limited, with most patients referred receiving one or two appointments before being discharged to GPs who have limited specialist training in management of people with cognitive impairment [13]. A review by Greenway-Crombie, Snow et. al. [14] highlighted the barriers to dementia diagnosis in rural general practice, which include a lack of specialist dementia support, stigma and negativity, diagnostic uncertainty and disclosure, lack of dementia-specific training, and the service complexity.

While the iterative process of GP education is essential, many GPs indicate a desire for specialist consultation in the areas of dementia diagnosis and management [15]. In Australia the need for referral is reinforced by the current prescription regulations which only allow for a pharmaceutical benefits scheme subsidy for dementia-specific medications if the initial prescription was by a medical specialist (eg geriatrician, neurologist or psychiatrist) or by a GP in consultation with such a specialist [16].

The program described in this paper sought to increase GP access to specialist advice, both through the co-location of a geriatrician and psycho-geriatrician specialists consulting within the primary setting, and through informal as-needed dialogue either in person or on the telephone. The program was also planned to remove barriers to patient attendance for dementia screening, by locating this within the general practice location, which was familiar and provided adequate free parking, and with no financial disincentive by funding specialist consultations through the Medicare program. The new model of care described in this paper was developed in a multi-site regional and rural general practice and aims to incorporate training and the use of standardised tools within primary care to address the limited access to screening and management of dementia in the community setting.

## Methods

### Study setting and sample

St Anthony Family Medical Practices P/L (SAFMP) are general practice-based group in the rural and regional, Loddon-Mallee region of Victoria, Australia. The area has a catchment of 12,213 km^2^ with 58,602 persons aged over 65 years, equivalent to 18.5% of the total population [17]. The SAFMP group encompassed seven practices, employing 48 general clinicians and providing 3,386 encounters for patients aged over 65 each year (2015 practice data).

### Model design: GP-Led early detection of cognitive impairment

To address access for people with cognitive impairment living in regional Victoria, a community-based high-quality diagnostic and management service model was developed. This included the education of GPs and PNs and the development of an early dementia detection screening and referral pathway to a community-based specialist clinic, the St Anthony Memory Service (Table 1).

**Table 1:** Model components

The GP-led early detection and screening for cognitive impairment model addressed many of the barriers established in the literature, by including:
1. A locally-based education program for the GPs and PNs working in the multi-site practice, to develop their knowledge of the spectrum of cognitive impairment, the use of screening tests in the diagnosis of cognitive impairment (and associated depression), and the essential clinical examination, laboratory and radiological investigation required as a baseline prior to referral;
2. Integration of routine screening, using validated tools, within existing practice systems and software;
3. Cognitive screening by GPs and PNs of all patients over 65 years who attended the practice, as well as those younger people who were concerned (or the GPs concerned) about their cognition;
4. The professional staff of the Memory Clinic included a Consultant in Geriatric Medicine, and a Psycho-Geriatrician; This allowed easy access to specialist support without the patient needing to attend a tertiary care institution for further management;
5. Communication with the GPs and PNs after each assessment, including written report and if necessary a case conference with the GP and PN via telephone or video;
6. If deemed appropriate by the GP, people identified with either Mild Cognitive impairment or Dementia (*referred to in ICD 11 as Minor and Major Neurocognitive Disorders)* were reviewed by the geriatrician or psychogeriatrician as frequently, and for as long as considered necessary thereafter

The model included: 1) the education of GPs and PNs; 2) the development of an early dementia detection screening and referral pathway; 3) provide access to a specialist cognitive assessment service based in the practice community; 4) raise awareness in the practice community about dementia and dementia risk management strategies; and 5) collect a body of patient data from initial screening through to cognitive assessment outcomes.

### Staff training

All practice group staff underwent a one-day training program in the delivery of cognitive assessments; including education on: recognising dementia in general practice; risk factors and management; use of treatments – pharmacological and non-pharmacological; behavioural and psychological symptoms of dementia (BPSD) and depression; caregiver stress, support services and respite; legal issues; end of life management, advanced directives and palliative care. Individual practices were offered support to provide community information sessions on the initiative, and on dementia and dementia risk minimization. An information sheet about dementia and the initiative was developed and placed in practice waiting rooms and given to those who underwent screening. GPs who attended voiced an increase in approaching people aged over 65 in regards to cognition screening as well as an increased confidence in the management of dementia in the primary care setting.

### Cognitive assessment process

Cognitive assessment was undertaken on all patients aged over 65 years who attended the GP practices, as well as patients who expressed concern regarding cognitive deficits, or patients who had concerns raised by an associated family member or caregiver. Practice nurses held an integral part of the process, doing the cognitive assessments and forming an essential part of the therapeutic relationship. Practice nurse screening often formed part of a routine 75 + assessment carried out by the nurses in the course of their usual practice. The assessment process consisted of: clinical and neurological examination by the GP; basic blood tests including full blood count, renal and hepatic function tests, assessment for diabetes, thyroid function, vitamin B_12_ and folate; brain CT scan; and cognitive assessment using Mini-Mental State Examination (MMSE) [18], GP Assessment of Cognition (GPCog) [19], and Geriatric Depression Scale (GDS) [20]. All participants with GPCog score of ≤ 9, and/or GDS ≥ 6 and/or MMSE ≤ 24 and/or those about whom the GP or PN was concerned, were referred to the project geriatrician or psycho-geriatrician (Table 2). The specialist service was based on a rapid response with no referred person waiting more than 4 weeks, and most seen within 2 weeks. Referral to other services included but was not limited to neuropsychologist, physiotherapy, occupational therapy and social work, and was done on an as needs basis as determined through the assessment.

**Table 2:** Dementia screen matrix and referral guide

Tool	**Score**			
GP Cog Pt 1	9	5–8	5–8	< 5
GDS	0–5	0–5	6–9	≥ 10
MMSE	≥ 25	21–24	21–24	≤ 20
Interpretation				
Likely outcome	No evident cognitive decline or depression	Possible cognitive impairment or mild dementia, no sign of depression	Possible cognitive impairment or mild dementia, and/or mild depression	Moderate/ severe depression with possibility of underlying moderate to severe dementia
Actions	Reassure patient and plan to review	Offer referral to geriatrician and arrange further investigations	Offer referral to geriatrician or psycho-geriatrician and arrange further investigations	Offer referral to psycho-geriatrician and arrange further investigations

### Data collection

Data was collected on all people screened between 2014 to 2018. Data collected included demographics, SEIFA (Socio-Economic Indexes For Areas) rank [21], medical history, smoking status, referral status and outcome, as well as GDS, GPCog, and MMSE scores.

### Statistical methods and data analysis

Data analysis was performed using SPSS statistical software version 26.0 (IBM Corp, Armonk, NY, USA). Patient demographics, medical history, and outcome scores are reported descriptively. Descriptive data is presented as mean and SD unless stated otherwise.

## Results

### Patient demographics

From the implementation of the model in 2014 to March 2017, 818 patients aged over 65 years were screened, accounting for approximately 24.2% of 65 and over encounters for the practice network (Table 3). Those screened had a mean age of 77.37 years (7.495 SD) and 51.1% (418) were female. The population was otherwise relatively homogenous, mainly born in Australia (611, 88.6%) and English speaking (> 99%). A third (275, 34.4%) noted that they lived alone.

**Table 3:** Patient demographics

Category (*n* = 818)	n	%
Age	Mean 77.37, SD 7.495 Range 41–99	
Gender reported		
Male	398	48.7
Female	418	51.1
Clinic		
Boort	83	10.1
Campaspe (Rochester)	102	12.5
Elmore	214	26.2
Heathcote	154	18.8
Spring Gully Primary Health	256	31.3
Waranga (Rushworth)	9	1.1
Country of birth (*n* = 690)		
Australian Born	611	88.6
Not Australian Born	79	11.4
Aboriginal and Torres Strait Islander (*n* = 557)		
No	554	99.5
Yes	3	0.5
Living alone (*n* = 799)		
Yes	275	34.4
No	524	65.6
Driving status (*n* = 761)		
Not driving	162	21.3
Driving	571	75.0
Restricted	28	3.7

Comprehensive relevant medical history was obtained in the majority of patients reviewed (Table 4). Three quarters (598, 75.4%) had a history of cardiovascular disease and half (431, 53.3%) were on statin therapy. Depression or anxiety and antidepressant use was reported in one in five patients. The majority (598, 90.1%) had no reported family history of dementia. One in ten had a history or stroke, brain, or head injury of various aetiology.

**Table 4:** Patient medical history

Medical History	n	%
Body mass index (BMI) (*n* = 765)	Mean 28.54, SD 5.127	Range 17.1–47.6
Diabetes (*n* = 772)		
No	609	78.9
Yes	163	21.1
Stroke, brain, or head injury (*n* = 805)		
None	724	89.9
Stroke	46	5.7
TIA	17	2.1
Subdural haemorrhage	5	0.6
Subarachnoid haemorrhage	2	0.2
Other brain injury	5	0.6
Noted as yes to head injury only	6	0.7
Cardiovascular (*n* = 793)		
No	195	24.6
Yes	598	75.4
Depression and anxiety (*n* = 795)		
None	646	81.3
Depression	124	15.6
Anxiety	25	3.1
Antidepressant usage (*n* = 810)		
No	633	78.1
Yes	177	21.9
Sleeping tablet usage (*n* = 808)		
No	673	83.3
Yes	135	16.7
Statin usage (*n* = 809)		
No	378	46.7
Yes	431	53.3
Alcohol intake (*n* = 685)		
None	287	41.9
< 2 std drinks/day for any given day	306	44.7
> 2 std drinks/day for any given day	92	13.4
Smoker status (*n* = 801)		
Never	464	57.9
Current	42	5.2
Ex-smoker	295	36.8
Hearing impairment (n = 550)		
No	317	57.6
Yes	233	42.4
Family history of dementia (n = 664)		
No	598	90.1
Yes	66	9.9

### Cognitive assessment

GDS was suggestive or indicative of depression in 13.8% of patients screened (Table 5). As expected, GDS suggestive of depression was significantly associated with formal diagnosis of depression (*p* < 0.001) and use of antidepressants (*p* < 0.001), but was also associated with a history of stroke, brain, or other head injury (*p* < 0.001), current smoker status (*p* < 0.001), and use of sleeping tablets (*p* = 0.004). Half (453, 55.9%) of patients screened using GPCog required additional testing to determine cognitive impairment whilst one in ten (82, 10.1%) had symptoms indicative of impairment. GPCog score indicating referral was significantly associated with diagnoses of depression or anxiety (*p* = 0.008) and use of antidepressants (*p* = 0.003). The majority (606, 87.4%) of patients showed no symptoms consistent with dementia on the MMSE outcome. MMSE scores indicative of dementia were significantly more frequent in men (*p* = 0.026) and people with diabetes (*p* = 0.042).

**Table 5:** Cognitive assessment scores

Assessment	n	%
**GDS** (*n* = 736)		
No depression	634	86.1
Suggestive of depression	84	11.4
Depression almost always present	18	2.4
Score	Mean 2.82, SD 2.661	Range 0–15
**GPCog** (*n* = 811)		
No significant cognitive impairment	276	34.0
More information required, proceed to further testing	453	55.9
Cognitive impairment indicated	82	10.1
Score	Mean 7.29, SD 1.968	Range 0–9
**MMSE** (*n* = 693)		
No dementia indicated	606	87.4
Suggestive of mild dementia	59	8.5
Suggestive of moderate dementia	27	3.9
Indicative of severe dementia	1	0.1
Score	Mean 27.40, SD 2.930	Range 3–30

### Referral rates

Of the 818 screened using at least one assessment tool, 564 satisfied the criteria for referral to a geriatrician and/or psycho-geriatrician as per the referral guide (Table 6), and 30.3% of these were successfully referred. Over half of the people who satisfied referral criteria (302, 53.5%) were not referred, and of the 260 patients referred, a third (89, 34.2% of referred patients) declined a specialist appointment. Of the 302 people who were not referred, the majority (273, 90.4%) had GPCog scores categorised as ‘more information required’. Similarly, most who were considered appropriate but declined referral (66, 74.2%) had GPCog scores categorised as ‘more information required’.

**Table 6:** Eligibility and actual referrals

			Indicated for referral	
			No@ (*n* = 254, 31.1%)	Yes@ (*n* = 564, 68.9%)
Outcome	Not referred	No referral attempted	249 (98.0%)	302 (53.5%)
		Already seeing specialist	0 (0.0%)	2 (0.4%)
	Referred	Declined	2 (0.8%)	89 (15.8%)
		Geriatrician	2 (0.8%)	135 (23.9%)
		Psycho-geriatrician	1 (0.4%)	34 (6.0%)
		Geriatrician/Psycho-geriatrician	0 (0.0%)	2 (0.4%)

## Discussion

*The National Framework for Action on Dementia 2015–2019* [22] highlights the challenges that people with cognitive impairment face in rural and regional areas in accessing appropriate medical management, including access to primary and specialist medical care. The current study was successful in conducting validated screening tests on one in four patients aged over 65 who presented at the St Anthony Family Medical Practices, the majority of whom were unlikely to be formally assessed prior to the program. Of those screened, two-thirds (68.9%) warranted further specialist investigation. These results highlight the opportunity of GPs to initiate early screening and intervention and are consistent with the National Framework emphasis on timely diagnosis and flexible modes of delivery [22]

Primary care in Australia is increasingly taking on a greater role in both the assessment and the long term care of people with dementia and a multicenter randomised controlled trial found no evidence that specialist memory clinics were more effective than general practice services in providing post-diagnostic support [23]. Secondary referral services, however, do have a critical role in defining the dementia subtype, dealing with more complex cases, and stratifying which patients with mild cognitive impairment are at greatest risk of developing dementia and most in need of follow-up. The primary motivation for developing the program described in this paper was to improve the local services for people with cognitive deficits, through encouraging the early identification of dementia and mild cognitive impairment by GP services and increasing access to specialist assessment for optimal management in the hope of modifying the progression of this through improved diagnosis, lifestyle changes and medication where appropriate.

Positive screening in the current study resulted in 171 successful referrals to a geriatrician and/or psycho-geriatrician. Nevertheless, it was disappointing that over half of the people who satisfied referral criteria were not referred, and a third of referred patients declined a specialist appointment. The majority of those who were either not referred, or declined a referral, had returned intermediate scores on the GPCog assessment which indicated a need for further assessment but did not necessarily indicate dementia. When compared to the MMSE, the GPCog has been shown to have increased sensitivity [24]. A study of 3750 geriatric patients previously identified 68% as requiring further testing, and of those 49% went on to have a diagnosable dementia [25]. Of the ‘false positives’ in that study, 38% showed signs of definitive cognitive impairment that did not meet diagnostic criteria [25]. These findings suggest that those with mild signs of impairment are most likely to be deemed as not requiring further care as well as more likely to reject specialist support. Efficacy of office-based screening for early dementia has indeed been called into question given limited treatment options and the likelihood of false positives causing unnecessary distress for patients [26]. Although referral rates in the current study are indicative of barriers to acceptance from either GPs or patients themselves which elicits further research on improving acceptability, the use of GPCog in general practice settings represents a valuable opportunity to detect and monitor cognitive impairment in geriatric populations.

Several factors drove the impetus for the development of this program, including informal discussions with local GPs which drew attention to the difficulties they faced in managing people with cognitive impairment, including limited confidence in the diagnosis of dementia and with disclosing the diagnosis to affected people and their family [15]. Practitioners reported difficulties in the referral process of affected people in this regional area, which had only one Memory Clinic with limited resources, and also noted reluctance of affected people to undergo specialist review for a problem that they were often reluctant to acknowledge as such [15].

These barriers are not unique to the target practice but have been echoed in broader regional and rural communities. Koch and Iliffe [6] reported that many barriers exist at the patient, doctor, and system level for a timely diagnosis, including the eradication of dementia-associated stigma in order to improve the early detection and management of dementia, while Bradford et. al. [7] identified issues with attitudes and patient-provider communication, educational deficits, and system resource constraints as major contributory factors.

A limitation of this program was that people who attend this practice form a relatively homogenous group, mostly born in Australia and English speaking, which is consistent with the demographics of regional Victoria. Whether the program would have been as acceptable to people of a more diverse ethnic background needs to be explored further in other settings if a program such as this is to be replicated across other primary health care settings. In addition, the long-term benefits of early detection and management of people with mild cognitive impairment and dementia also needs to be investigated in the long term in parallel to upscaling the capacity of the Australian health system to manage the increased demand.

## Conclusions

Dementia screening can be offered to all patients aged 65 and over in rural settings when integrated as a model of GP-led screening with referral pathways to community-based specialist services. Implementation of standardised processes and practitioner training markedly improved early screening and referral for patients with cognitive change. This new, innovative model of care is scalable across health systems with potential to address geographical barriers through online screening and engagement. Future research should aim to explore interventions to support management of cognitive impairment for people living with dementia in the rural setting.

## Data Availability

The datasets generated and/or analysed during the current study are not publicly available due to obtained consent to publish only aggregate and de-identified data but are available from the corresponding author on reasonable request.

## Abbreviations

BMI: Body mass index
BPSD: Behavioural and psychological symptoms of dementia
GDS: Geriatric depression scale
GP: General practitioner
GPCog: General practitioner assessment of cognition
MMSE: Mini-mental state examination
SAFMP: St anthony family medical practices
SEIFA: Socio-economic indexes for areas
